# Deep learning NTCP model for late dysphagia after radiotherapy for head and neck cancer patients based on 3D dose, CT and segmentations

**DOI:** 10.1016/j.radonc.2025.111169

**Published:** 2025-09-29

**Authors:** S.P.M. de Vette, H. Neh, L. van der Hoek, D.C. MacRae, H. Chu, A. Gawryszuk, R.J.H.M. Steenbakkers, P.M.A. van Ooijen, C.D. Fuller, K.A. Hutcheson, J.A. Langendijk, N.M. Sijtsema, L.V. van Dijk

**Affiliations:** a Department of Radiotherapy, University Medical Center Groningen, University of Groningen, Groningen, the Netherlands; b Department of Radiation Oncology, the University of Texas MD Anderson Cancer Center, Houston, USA; c Department of Head and Neck Surgery, the University of Texas MD Anderson Cancer Center, Houston, TX, USA

**Keywords:** Dysphagia, Normal Tissue Complication Probability, Deep learning, Head and neck cancer, Risk prediction, Radiotherapy, Radiation-induced toxicity

## Abstract

**Background & purpose:**

Late radiation-associated dysphagia after head and neck cancer (HNC) significantly impacts patient’s health and quality of life. Conventional normal tissue complication probability (NTCP) models use discrete dose parameters to predict toxicity risk but fail to fully capture the complexity of this side effect. Deep learning (DL) offers potential improvements by incorporating 3D dose data for all anatomical structures involved in swallowing. This study aims to enhance dysphagia prediction with 3D DL NTCP models compared to conventional NTCP models.

**Materials & methods:**

A multi-institutional cohort of 1484 HNC patients was used to train and validate a 3D DL model (Residual Network) incorporating 3D dose distributions, organ-at-risk segmentations, and CT scans, with or without patient- or treatment-related data. Predictions of grade ≥ 2 dysphagia (CTCAEv4) at six months post-treatment were evaluated using area under the curve (AUC) and calibration curves. Results were compared to a conventional NTCP model based on pre-treatment dysphagia, tumour location, and mean dose to swallowing organs. Attention maps highlighting regions of interest for individual patients were assessed.

**Results:**

DL models outperformed the conventional NTCP model in both the independent test set (AUC = 0.80–0.84 versus 0.76) and external test set (AUC = 0.73–0.74 versus 0.63) in AUC and calibration. Attention maps showed a focus on the oral cavity and superior pharyngeal constrictor muscle.

**Conclusion:**

DL NTCP models performed significantly better than the conventional NTCP model, suggesting the benefit of using 3D-input over the conventional discrete dose parameters. Attention maps highlighted relevant regions linked to dysphagia, supporting the utility of DL for improved predictions.

## Introduction

Dysphagia is a common late side-effect of radiotherapy for head and neck cancer (HNC), significantly impairing patients’ quality of life by limiting intake [1]. Furthermore, dysphagia may increase the risk of aspiration and consequently aspiration pneumonia, a potentially life-threatening disease [2]. Normal tissue complication probability (NTCP) models can estimate the risk of developing dysphagia following radiotherapy and are typically based on discrete organ at risk (OAR) dose values and/or patient characteristics [3,4]. Radiation-associated dysphagia (RAD) risk estimation can be used to guide dose optimization and treatment decision-making, such as selecting patients for either photon or proton therapy [5,6].

Swallowing is a complex mechanism, involving over 30 muscles and nerves that collaborate to move ingesta from mouth to stomach [7–9]. Current NTCP models predicting late RAD include dose-volume parameters of pharyngeal constrictor muscles (PCMs) [3,4], either combined with the supraglottic larynx [3], oral cavity [4] or brainstem [10]. However, this is a limited representation of swallowing OARs [11–13]. Including dose parameters of all relevant swallowing muscles in the conventional logistic regression NTCP models can be challenging due to multicollinearity (i.e. high correlation between parameters) and overfitting (i.e. creating a complex model that does not generalise to new patients) [14]. Furthermore, NTCP models often rely on mean doses or dose-volume thresholds to specific OARs, which may oversimplify dose distributions, losing important spatial details [13]. Hence, more advanced modelling is needed to analyse complex radiation dose data by extracting information from 3D dose distributions and imaging information of all swallowing-related regions.

Deep learning (DL) methods can process complete 3D dose distributions and CT scans to predict toxicity risk, without reducing radiation dose to single values (1D) as in conventional NTCP models. Furthermore, by leveraging comprehensive anatomical information from CT scans and radiation dose distributions, DL models can identify complex relationships between radiation dose and all OARs visible on the CT scan. This represents additional advantages over conventional logistic regression NTCP models, as it eliminates the need for manual model parameter selection and better handles multicollinearity by reducing overlapping information between related input factors [15,16]. Moreover, clinical variables such as tumour location and pre-existing conditions (e.g. baseline dysphagia) can be incorporated in both conventional and DL models to provide all relevant information for the prediction of late toxicities [2,4,17,18]. For xerostomia (i.e. dry mouth), DL models have already demonstrated superior performance compared to conventional NTCP models and highlight sub-regions of the parotid glands as important structures for xerostomia prediction [19,20]. This DL approach may be particularly suitable for predicting late RAD, given the interconnected nature of swallowing muscles, the complex damage mechanisms involved, and the variety of data that can be used for predicting RAD (e.g., pre-treatment factors, CT scans, and dose distributions).

Therefore, the aim of this study was to improve the prediction of RAD at six months after treatment with DL compared to a validated conventional NTCP model for HNC patients. Additionally, this study explored multiple methods to incorporate clinical variables in a DL model.

## Methods and materials

### Patient data

Patients were treated at the University Medical Centre Groningen (UMCG, 2007–2021) and MD Anderson Cancer Centre (MDACC, 2015–2021). Patient symptom, tumour, clinical and treatment data were collected prospectively as part of active standardized follow-up registry studies approved by the respective hospital Institutional Review Boards (UMCG: NCT02435576, MDACC: PA14–0947 (data collection) and PA11–0809 (data analysis)), in which patients gave consent to the use of their data. Patients met the following inclusion criteria: 1) squamous cell carcinoma of the head and neck region (excluding skin), 2) treated with definitive curative HNC radiotherapy, 3) finished all planned treatment fractions, 4) dysphagia assessment available pre-treatment and 6 months after RT, 5) were ≥ 18 years, 6) did not have previously treated HNC or surgery in the head and neck region (except tonsillectomy or laser treatment for small glottic lesions).

The UMCG cohort was used for DL model development (85 %: 70 % training, 15 % internal validation) and independent testing in a never-seen-by-the-model set (15 %). This split was stratified by treatment modality (photon- vs proton-treatment), CT contrast-enhancement, and CT artefacts. The MDACC cohort served as external validation.

UMCG patients received 66–70 Gy to the primary tumour and 54.25 Gy as elective dose in 33–35 fractions with or without concurrent platinum-based chemotherapy or cetuximab. MDACC patients received 66–70 Gy to the primary tumour and 56–57 Gy as elective dose in 30–33 fractions with or without concurrent platinum-based chemotherapy. In both centres, salivary glands were spared as much as possible during treatment optimisation. Additionally, starting in 2010, swallowing structures were also spared at UMCG [21]. Refer to supplementary materials A for swallowing-sparing radiotherapy techniques.

Planning CT-scans were acquired on which OARs were manually delineated following the guidelines of Brouwer et al. and Christianen et al. (PCMs only) for the UMCG cohort [22,23], while Atlas-based auto-contouring algorithm by Elekta ADMIRE was used in the external MDACC cohort [24]. Missing OAR segmentations were supplemented by the clinically deployed Deep Learning Contours software [25]. In previous studies, the Deep Learning contours were benchmarked against manual contours [25], and the mean doses from the Atlas-based auto-contours deployed at MDACC the MDACC were compared to the Deep Learning contours [26]. Refer to supplement B for CT parameters. Dose-volume histogram parameters were extracted from the dose distributions and OAR segmentations using MATLAB (version R2018b). Patient data from the external MDACC cohort can be found at: https://doi.org/10.6084/m9.figshare.29608733.

### NTCP endpoint

At UMCG, physician-rated dysphagia was scored using the adjusted Common Terminology Criteria for Adverse Events (CTCAEv4), with grade 2 (symptomatic and altered eating/swallowing) or higher at six months after radiotherapy as the NTCP endpoint. At MDACC, clinician-rated dysphagia was assessed using the “diet normalcy” question from the Performance Status Scale for Head and Neck cancer (PSS-HN), with scores of ≤ 60 at 3–6 months after radiotherapy defined as the NTCP endpoint, as this best corresponded to CTCAE grade 2 dysphagia [26]. The median time at which the PSS-HN was assessed was 135 days (4.5 months, inter quartile range = 99–162 days) post-radiotherapy. Refer to supplement C for toxicity grading scales.

### Reference NTCP model

This study used as a reference the conventional moderate-to-severe RAD NTCP model published by Van den Bosch et al., which has been validated twice [4,26]. The model includes the mean dose to the oral cavity, the inferior, middle, and superior PCM, pre-treatment dysphagia score and tumour location [4]. Logistic regression coefficients were recalculated in each training fold (see supplement D), to make this NTCP model the most competitive comparator for the DL model. Performance was assessed on the independent and external test set.

### Deep learning (DL) model architecture and training

A Residual Network (ResNet) was used as DL architecture to predict RAD from 3D dose distributions, CTs and OAR segmentations [27]. The Tree-structured Parzen Estimator algorithm in the open-source Optuna framework guided the fine-tuning of the model’s settings (hyperparameters) [28]. Differences in model architecture (e.g. number and size of linear layers), hyperparameters (e.g. learning rate, scheduler, batch size), and data augmentation strategies (e.g. amount of data augmentation, use of AugMix [29]) were tested. Ultimately, the optimal hyperparameters were selected based on the mean validation loss during 5-fold cross-validation. The data augmentation and modelling were implemented using Project MONAI 0.8 in PyTorch 1.13 [30,31]. For more details on model training and preprocessing refer to supplementary E. The source code has been made publicly available at https://github.com/PRI2MA/DL_NTCP_Dysphagia.

**Fig. 1.**
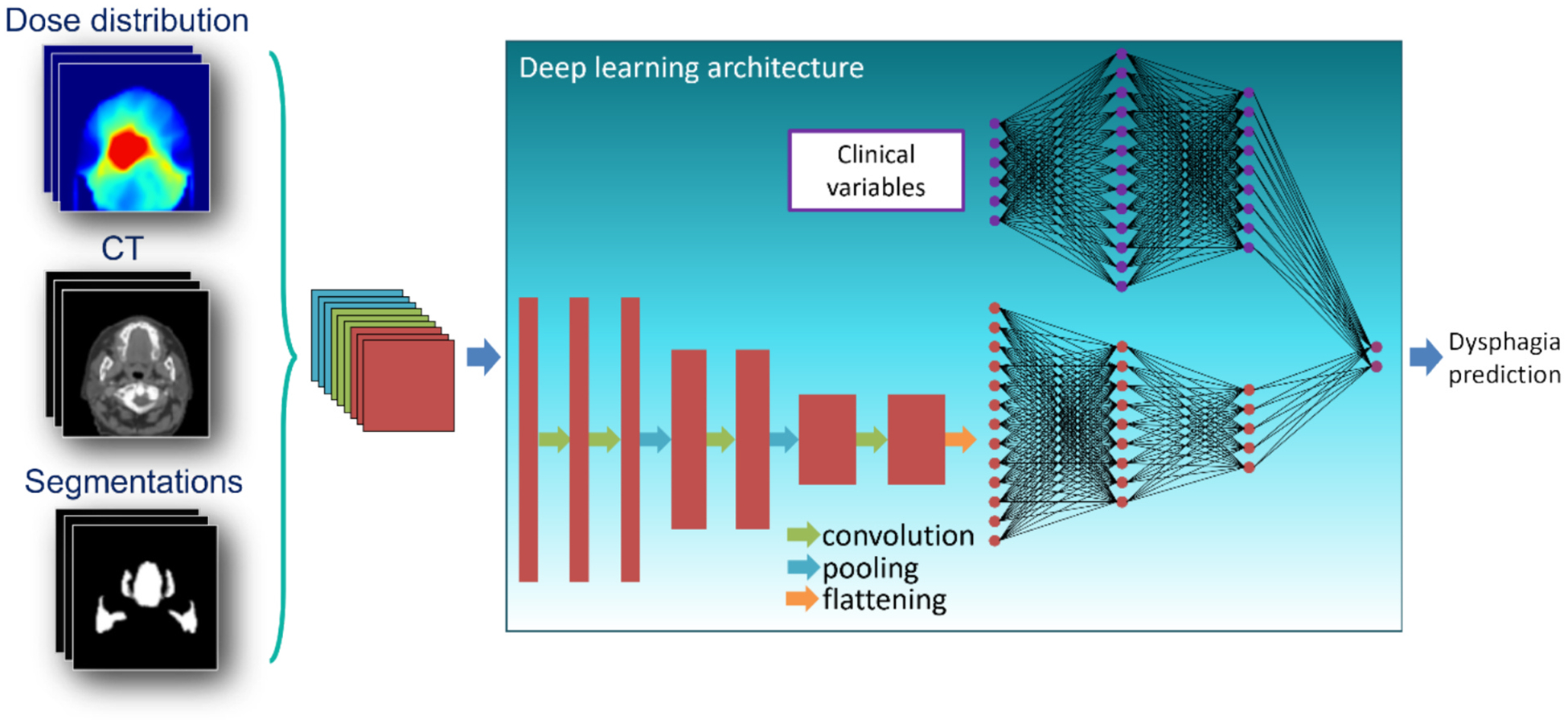
General model architecture of deep learning model. 3D DL-input of the model is the dose distribution, CT and segmentations (left) and 1D DL-input of the model is an array of patient- and treatment related (clinical) variables (middle top).

A schematic overview of the model structure is depicted in Fig. 1. The DL RAD risk prediction had two types of input: the pre-processed 3D CT-scan, 3D dose distribution and 3D OAR segmentations (hereafter called ‘3D DL-input’) and clinical variables (hereafter called ‘1D DL-input’). The 1D DL-input includes dysphagia-related variables (pre-treatment dysphagia score, pre-treatment xerostomia score, tumour location) and commonly reported patient- and treatment-related variables (T-stage, N-stage, smoking status prior to treatment, HPV-P16 status and pre-treatment WHO performance score). Multiple model configurations were tested, which are outlined in supplement F. Models were created based on only the 3D DL-input (Fig. S1A: ResNet_3D_) or both the 3D and 1D DL-inputs (Fig. S1B: ResNet_3D+1D(LR)_ and C: ResNet_3D+1D_). In ResNet_3D+1D(LR)_, two models (one based on 3D DL-input and one on 1D DL-input) are optimized separately, after which their predictions are combined using logistic regression (LR), whereas in ResNet_3D+1D_, only one model is trained and the balance between 3D and 1D DL-input is optimized simultaneously with the rest of the model.

### Model evaluation

Final models were evaluated using the area under the receiver-operator curve (AUC), calibration plots, Nagelkerke R^2^, and the Brier score. Metrics were based on ensemble predictions for the independent and external test cohorts, and averaged across 5 folds for internal validation. DL models were compared to the reference logistic regression NTCP model with updated weights and tested in the same cohorts.

### Attention maps

Attention maps are visual tools that highlight the most important areas in an image influencing a model’s decision. They use the model’s weights and the 3D DL-input of each individual patient to create personalised visualisations. In this study, they show anatomical regions contributing most to NTCP values for individual patients. Attention maps were generated using Grad-CAM++ for the ResNet_3D_ model [32]. Attention maps were generated in the independent test cohort for each separate cross-validation fold. They were visually evaluated to identify the regions within the head and neck area that contribute most to the prediction of RAD. Additionally, the mean attention per OAR was calculated.

### Contribution of model inputs

The individual contribution of the 3D DL-inputs was evaluated by performing a leave-one-out analysis on the DL NTCP models trained with all 3D DL-inputs. Instead of the true input (dose, segmentations or CT), a blank input was used to assess the impact of omitting the dose, segmentations or CT on the calibration plots. Furthermore, to assess the influence of clinical variables on the final DL model, we synthetically varied one clinical variable at a time for all test patients while keeping all other clinical variables constant and evaluating the change in predicted NTCP.

## Results

**Fig. 2.**
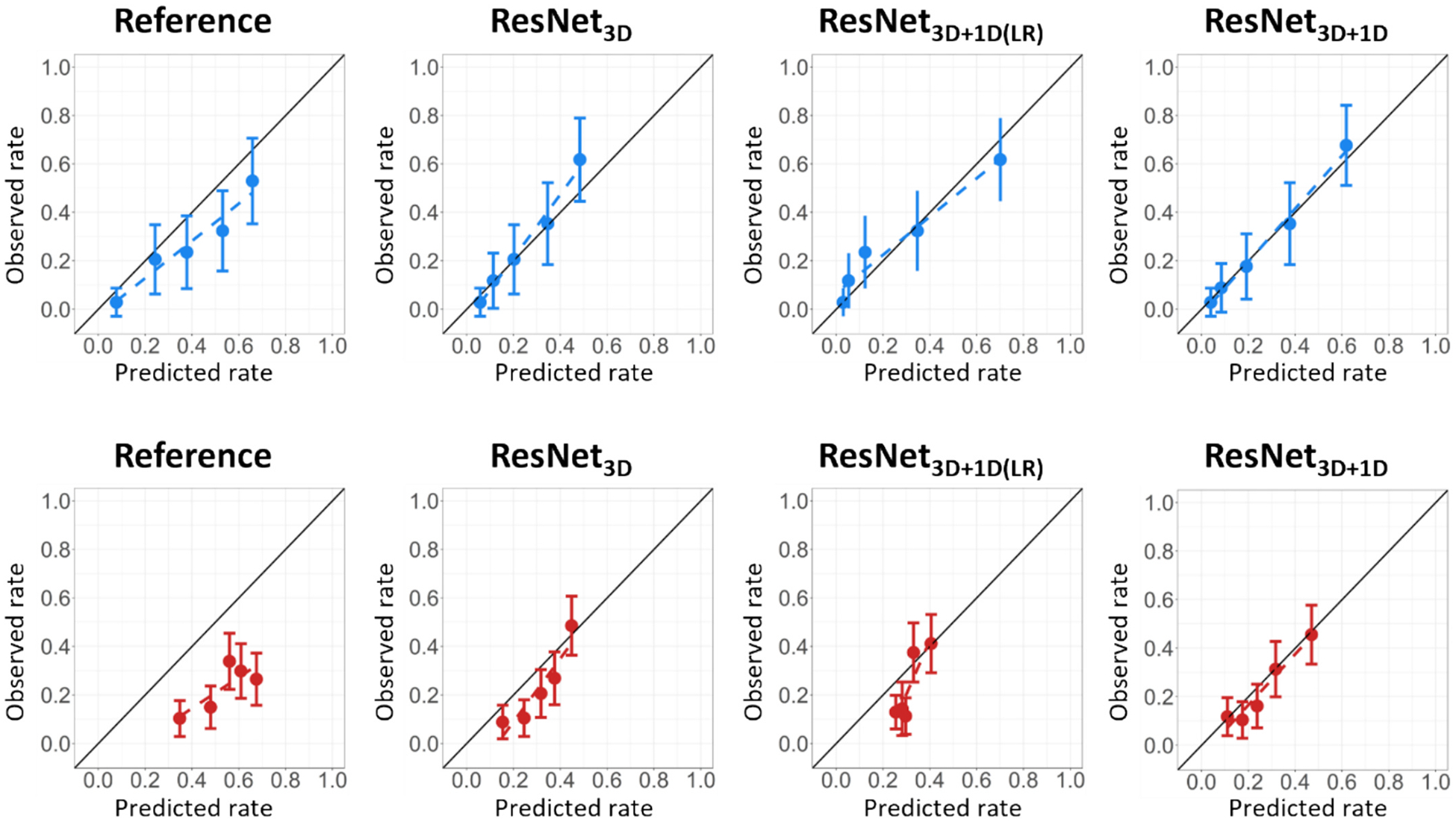
Calibration plots of model performance on independent test cohort (blue, top) and external test cohort (red, bottom).

The final UMCG cohort included 1112 HNC patients, split in 941 for training and internal validation and 171 for independent testing (refer for patient exclusions to supplement G). The external MDACC test cohort consisted of 338 patients. RAD event rates six months after treatment were 25 % (training and internal validation), 26 % (independent testing), and 23 % (external testing). Patient characteristics did not differ between the UMCG train and test cohorts, yet were significantly different for UMCG patients and MDACC patients (Table 1). The higher proportion of ‘unknown’ P16 status in the UMCG cohort compared to the MDACC cohort reflects institutional practice, as only oropharyngeal cancer patients are routinely tested for P16 status at the UMCG. The final hyperparameters yielding the lowest validation loss are specified per model in supplement H. All DL ResNet NTCP models outperformed the reference NTCP model across all cohorts in AUC, R^2^, and Brier score (Table 2), as well as calibration (Fig. 2). In the independent test cohort, ResNet_3D_ achieved a higher AUC (AUC = 0.80 [Confidence interval = 0.73–0.88]) compared to the reference model (0.76 [0.68–0.84]). The performance gain was more pronounced in the external test cohort, where the ResNet_3D_ achieved an AUC of 0.73 [0.66–0.79] versus 0.63 [0.56–0.69] for the reference model, although the overall performance decreased for all models.

The best overall performance was observed when incorporating both the 3D and 1D DL-inputs. In the independent test cohort, ResNet_3D+1D(LR)_ achieved an AUC of 0.83 [0.75–0.90], and the ResNet_3D+1D_ reached 0.84 [0.77–0.90], which was significantly better than the reference model (p ≤ 0.05, DeLong’s test). However, the 1D DL-inputs did not improve the performance in the external test cohort, with the ResNet_3D+1D(LR)_ achieving an AUC of 0.74 [0.68–0.80] and the ResNet_3D+1D_ an AUC of 0.73 [0.66–0.79]. All DL models showed a statistically significant (p ≤ 0.05, DeLong’s test) improvement over the reference model in the external test cohort.

The R^2^ indicated that the ResNet_3D+1D_ had the best goodness-of-fit of all models with values of 0.39 and 0.16 in the independent and external test cohorts, respectively. Furthermore, this model also obtained the lowest Brier scores (0.14 and 0.16 in the independent and external test cohorts, respectively), indicating the best accuracy among these models.

Fig. 2 shows that the model calibration improved in all ResNet models compared to the reference model in both the independent and external test cohort. The best calibration was achieved with ResNet_3D+1D_, demonstrating that the predicted rate closely aligns with the observed RAD rate.

**Fig. 3.**
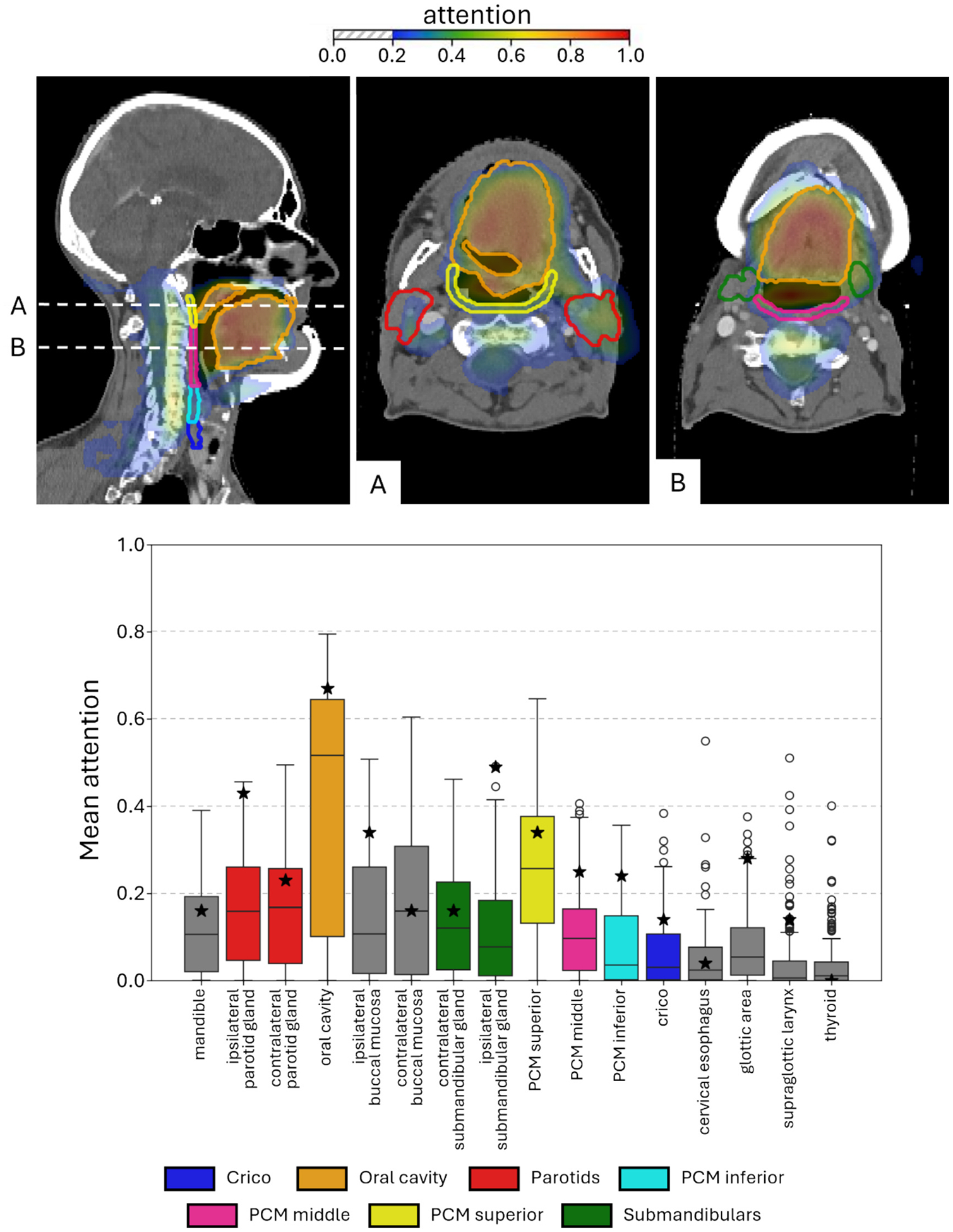
Attention maps. Top: attention map of a patient with high predicted probability for developing dysphagia, indicating that the oral cavity (including hyoglossus and styloglossus), PCMs and salivary glands are regions that contribute to the prediction of dysphagia. Panel A and B are slices from height A and B. Bottom: box plot of mean attention to several OARs. The star indicates the mean attention in the OAR in the attention map of the patient at the top. Abbreviations: OAR = organ at risk, PCM = pharyngeal constrictor muscle.

An attention map for a patient who developed RAD after treatment is depicted at the top of Fig. 3. It shows that for this patient, the oral cavity, PCMs and salivary glands were the most important regions that contribute to the prediction of RAD at 6 months after treatment. As attention maps differ per patient, the mean attention to OARs over all patients in the independent test cohort is shown at the bottom of Fig. 3. This panel shows that the oral cavity and PCM superior received the most attention across the cohort.

**Fig. 4.**
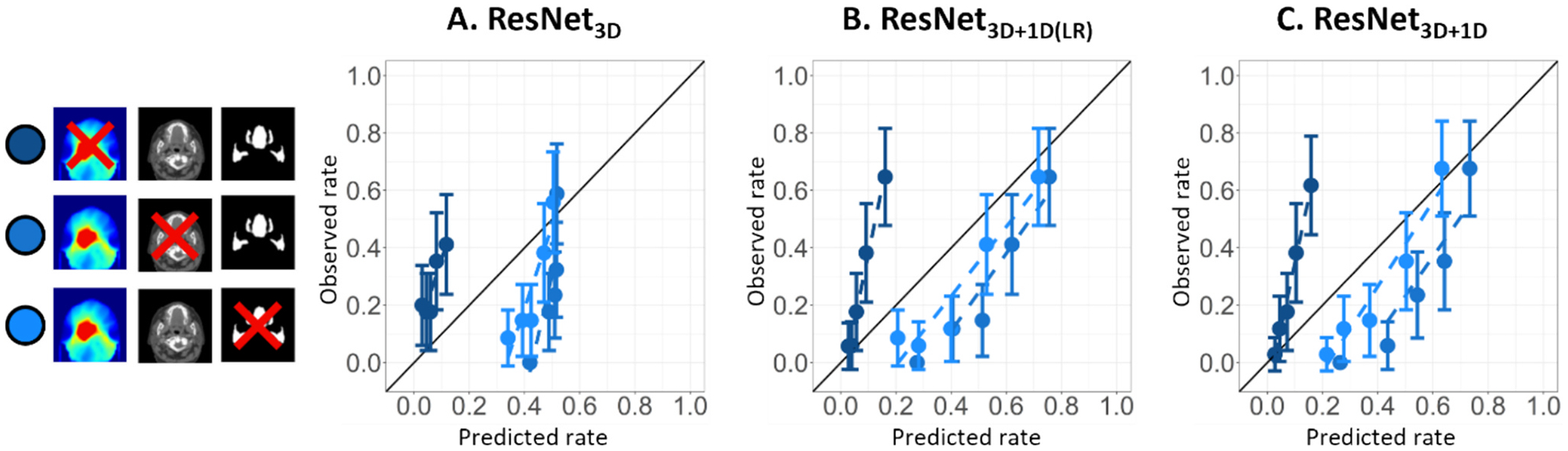
Calibration plots of leave-out-analysis in the independent test set. Darkest shade of blue: no dose, middle shade of blue: no CT, lightest shade of blue: no segmentations. Abbreviations: ResNet = Residual network.

Omitting any 3D input modality affected the model’s calibration performance (Fig. 4). Omitting dose data led to overall lower predictions, producing a steep calibration curve. Furthermore, omitting segmentations resulted in higher predictions, and the spread in predictions became smaller for both ResNet_3D_ and ResNet_3D+1D_ (Fig. 4A and C), though this effect was not observed in ResNet_3D+1D(LR)_ (Fig. 4B). When CT data was omitted, predictions from ResNet_3D_ clustered around 0.5 (Fig. 4A), while ResNet_3D+1D_ and ResNet_3D+1D(LR)_ showed a wider spread (Fig. 4B and C). The same analysis was performed on the external test cohort, with similar results (shown in supplement I). Synthetically varying between the lowest and highest category of the input clinical variables for the ResNet_3D+1D_ showed the largest influence for T-stage, P16 status, and pre-treatment dysphagia score, with average NTCP differences of 6 %, 5 %, and 4 %, respectively.

## Discussion

The 3D DL models substantially improved the prediction of RAD 6 months after radiotherapy for HNC patients compared to the 1D reference model in both an independent (AUC = 0.80–0.84 vs 0.76) and external test cohort (AUC = 0.73–0.74 vs 0.63). This suggests that DL models based on 3D dose distribution, CT, and segmentations capture more information regarding the development of RAD than the reference logistic regression NTCP model, which relied on 1D oral cavity and PCM substructures mean doses, tumour location and pre-treatment dysphagia score. As an additional reference, the widely used dysphagia prediction model by Christianen et al. (2012) [3] was evaluated, achieving an AUC of 0.69 [0.60–0.78] in the independent test cohort, and was thus outperformed by the DL models. Moreover, the addition of clinical variables improved the prediction of RAD even further in the independent test cohort (AUC = 0.83–0.84 vs 0.80).

Many swallowing OARs contribute to the complex process of safe and effective swallowing [11]. Since the DL model processes both anatomical and dose information, it should capture dose effects on all interrelated swallowing OARs. Gawryszuk et al., described the swallowing motion based on functional swallowing units (FSUs), demonstrating the importance of including more swallowing OARs in radiotherapy treatment planning and NTCP modelling for aspiration [33]. In the attention map of the individual patient (Fig. 3, top), the DL model focused on the oral cavity, including its most lateral component, the hyoglossus/styloglossus complex, an important FSU typically included within the oral cavity structure. A trend of elevated attention to the oral cavity and PCM superior was also observed in the complete independent test cohort (Fig. 3, bottom). Attention maps can highlight which areas the model considers important for its prediction, but they do not reveal how dose-alterations in these areas would affect the prediction [34]. As a future direction, perturbation-based methods could be applied to the 3D dose distribution, where the dose to specific regions is selectively synthetically increased or decreased to test how the model’s NTCP predictions change. This could identify dose-sensitive areas captured by the model and could ultimately guide treatment planning by prioritizing dose sparing in those regions.

Two methods for incorporating clinical variables in the model have been explored. In the first method, one model based on both 3D and 1D DL-input was created (ResNet_3D+1D_), while in the second method the output of two separate models (one based on 3D DL-input and one based on 1D DL-input) was combined with logistic regression (ResNet_3D+1D(LR)_). Both approaches improved performance compared to using imaging data alone (ResNet_3D_). While the differences in AUC were limited, the direct integration (ResNet_3D+1D_) showed more consistent calibration and better goodness-of-fit, particularly in the external test cohort. This aligns with expectations, as during model training, model weights for the parts processing 3D DL-input and 1D DL-input are optimized simultaneously, directly balancing all information from both DL-input types. In ResNet_3D+1D(LR)_, this balance is achieved by combining two separate models, meaning the 3D DL-input and 1D DL-input are processed independently. This prevents the model from establishing a specific relationship between, e.g. a dose parameter from the 3D DL-input and the pre-treatment dysphagia score from the 1D DL-input, which would be possible in ResNet_3D+1D_.

In the leave-one-out-analysis, omitting radiation dose from the 3D DL-input resulted in lower predictions compared to when all input was available (Fig. 4), aligning with the intuitive reasoning that no dose implies no expected toxicity. Omitting CT or segmentations from the 3D DL input caused risk overestimation in both test cohorts (Fig. 4 and Fig. S4), suggesting the model uses them to lower RAD predictions. Overall, this suggests that the DL models heavily rely on dose to swallowing OARs. As the absence of CT input had a modest effect on the predictions, it is unlikely that variation in contrast-enhancement of the CTs impacted the performance of the DL models.

As post-treatment RAD is related to damage to swallowing muscles, an insight into the pre-treatment structural status of these muscles may provide more information for the prediction of RAD [11]. In our modelling approach, CTs were used to incorporate information about anatomy, but muscle mass, and especially muscle quality, can be better determined by MRI than by CT, due to the improved soft tissue contrast [35]. Pre-treatment sarcopenia, a type of muscle loss that can be assessed using MRI, is related to the development of RAD after treatment [36]. Therefore, developing a model that contains MRI in the 3D DL-input would be promising to improve the prediction of RAD even further. Moreover, this study focused solely on developing a DL model predicting RAD 6 months after treatment, a time point during the transition from acute to late toxicity. Future work should aim to model the complete dysphagia trajectory, capturing this transition and predicting later RAD rates.

Before this model can be applied in clinical practice, we recommend an initial phase of parallel use alongside an established clinical model, such as that of Van den Bosch et al. (2021) [4]. Although the model meets the TRIPOD type 4 criterium through external validation, its lower interpretability compared to traditional logistic regression models warrants a more cautious implementation strategy [37].

One of the main limitations of this study was the significant difference in patient characteristics, tumour locations and endpoint assessments (CTCAE vs PSS-HN diet normalcy score) between the UMCG and MDACC cohorts, potentially influencing results in the external test cohort. In the MDACC cohort patients were also staged using both TNM version 7 and 8. This likely contributed to the lower performance of both the reference and DL models in the external test cohort. Nevertheless, the DL models still showed a large performance increase compared to the 1D reference NTCP model in the external test cohort. Additionally, FSUs were not delineated per guideline [38], as this approach is highly time-consuming, making it impractical for 1,484 patients. Consequently, they were not included as segmentations in the DL models, and the attention map analysis in the independent test set was limited to 16 OARs from the guidelines by Brouwer et al. [22]. Furthermore, dysphagia was essentially assessed using clinician-rated oral intake, a non-specific measure of function. In HNC, impaired oral intake reflects oral and pharyngeal dysfunction, but also salivary, taste, appetite, and pain symptoms. As such, more comprehensive and swallowing-specific imaging-based assessments such as Modified Barium Swallowing studies (MBS) are expected to yield more precise RAD-specific models [39]. MBS has been used in previous studies to assess how swallowing patterns are altered by radiation dose, which could offer a more accurate prediction of RAD based on dose [40,41]. In a future study, our 3D modelling approach could be combined with MBS-based outcome metrics, e.g. the DIGEST score [42], to improve RAD prediction by finding stronger relations between the affected swallowing structures and the dose that was delivered. Lastly, the size of our development cohort may be insufficient given the complexity of the DL model, as large, high-quality datasets are essential for robust model training and these cohorts are limited in the medical domain. Developing a DL model on larger multi-centre datasets could provide a solution to this problem.

## Conclusion

The 3D DL NTCP models predicting RAD after radiotherapy significantly outperformed the reference 1D NTCP model in both the independent and the external test cohort based on discrimination and calibration. Adding clinical variables to the 3D DL NTCP model improved the performance even further. These results suggest that RAD prediction can be improved by using the entire 3D dose distribution, OAR segmentations, the CT scan and clinical variables.

## Supplementary Material

Appendix A. Supplementary data

Appendix A. Supplementary data

Supplementary data to this article can be found online at https://doi.org/10.1016/j.radonc.2025.111169.

## Figures and Tables

**Table 1:** Patient characteristics.

	Training (UMCG) n = 941	Independent test (UMCG) n = 171	External test (MDACC) n = 338	P value (UMCG – MDACC)
***Age (mean (SD))***	64 (10)	64 (10)	61 (9)	<0.001^‡^
***Sex, No. (%)***				<0.001^†^
Male	710 (76)	125 (73)	309 (91)	
Female	231 (24)	46 (27)	29 (9)	
***Tumour location (%)***				<0.001^†^
Larynx	410 (44)	72 (42)	0 (0)	
Oral cavity	50 (5)	12 (7)	0 (0)	
Pharynx	468 (50)	85 (50)	338 (100)	
Other*	13 (1)	2 (1)	0 (0)	
***T stage***^¶^ ***(%)***				<0.001^†^
Tis-T2	452 (48)	80 (47)	234 (69)	
T3-T4	488 (52)	91 (53)	104 (31)	
***N stage***^¶^ ***(%)***				<0.001^†^
N0	443 (47)	76 (44)	25 (7)	
N1	86 (9)	20 (12)	124 (37)	
N2	383 (41)	73 (43)	182 (54)	
N3	29 (3)	2 (1)	7 (2)	
***P16-status (%)***				<0.001^†^
Positive	158 (17)	26 (15)	253 (75)	
Negative	215 (23)	39 (23)	15 (4)	
Not tested	568 (60)	106 (62)	70 (21)	
***Treatment technique (%)***				<0.001^†^
3D CRT	70 (7)	10 (6)	0 (0)	
IMRT	414 (44)	76 (44)	57 (17)	
VMAT	317 (34)	61 (36)	193 (57)	
IMRT + VMAT	9 (1)	2 (1)	22 (6)	
IMPT	131 (14)	22 (13)	66 (20)	
***Treatment modality***				<0.001^†^
With systemic treatment	377 (40)	70 (41)	262 (78)	
Without systemic treatment	564 (60)	101 (59)	76 (22)	
***Smoking status (%)***				<0.001^†^
Yes	447 (47)	75 (44)	1 (0)	
No	494 (53)	96 (56)	3 (2)	
Missing^§^	0 (0)	0 (0)	334 (98)	
***WHO score (%)***				<0.001^†^
WHO 0	652 (70)	115 (69)	0 (0)	
WHO 1	229 (25)	41 (25)	0 (0)	
WHO 2	47 (5)	9 (5)	0 (0)	
WHO 3	2 (0)	2 (1)	0 (0)	
WHO 4	1 (0)	0 (0)	0 (0)	
Missing^§^	0 (0)	0 (0)	338 (100)	
***Contrast CT available (%)***				<0.001^†^
Yes	767 (82)	144 (84)	0 (0)	
No	173 (18)	27 (16)	338 (100)	
***Baseline dysphagia (%)***				<0.001^†^
Grade 0–1	758 (81)	134 (78)	306 (91)	
Grade 2	118 (12)	27 (16)	30 (9)	
Grade 3–4	65 (7)	10 (6)	2 (0)	
***Dysphagia M6 (%)***				0.52^†^
Grade < 2	708 (75)	126 (74)	260 (77)	
Grade ≥ 2	233 (25)	45 (26)	78 (23)	
***Baseline xerostomia (%)***				<0.001^†^
None	522 (56)	87 (51)	234 (69)	
Little	292 (31)	59 (34)	49 (15)	
Moderate	68 (7)	14 (8)	25 (7)	
Severe	22 (2)	3 (2)	10 (3)	
Missing^§^	37 (4)	8 (5)	20 (6)	

Abbreviations: SD = standard deviation, 3D CRT = 3D Conformal Radiotherapy, IMRT = Intensity Modulated Radiotherapy, VMAT = Volumetric Modulated Arc Therapy, IMPT = Intensity Modulated Proton Therapy, M6 = 6 months after radiotherapy.

* Including nasal cavity, lacrimal gland and parotid gland.

‡ Kruskal-Wallis test.

† Chi-squared test.

¶ TNM staging version 7 for training and independent test cohort, TNM staging version 7 (n = 205) and 8 (n = 133) for external test cohort.

§ Missing values were assumed to indicate absence during modeling: non-smoker, WHO 0 and no baseline toxicity.

**Table 2:** Model performance.

	Cohort	Reference	ResNet_3D_	ResNet_3D+1D (LR)_	ResNet_3D+1D_
AUC [95 % CI]	Cross-validation	0.78 [0.75–0.81]	0.83 [0.80–0.86]	**0.85** **[0.82**–**0.88]**	0.84 [0.81–0.87]
	Independent test	0.76 [0.68–0.84]	0.80 [0.73–0.88]	0.83 [0.75–0.90]	**0.84** **[0.77**–**0.90]**
	External test	0.63 [0.56–0.69]	0.73 [0.66–0.79]	**0.74** **[0.68**–**0.80]**	0.73 [0.66–0.79]
R^2^	Cross-validation	0.141	0.330	**0.404**	0.384
	Independent test	0.148	0.286	0.358	**0.391**
	External test	−0.683	0.092	0.128	**0.164**
Brier score	Cross-validation	0.171	0.141	**0.129**	0.131
	Independent test	0.178	0.154	0.139	**0.135**
	External test	0.264	0.165	0.161	**0.155**

Abbreviations: AUC = Area Under the Curve, CI = Confidence Interval.

## Abbreviations:

AUC: Area under the curve
CTCAE: Common Terminology Criteria for Adverse Events
DL: Deep learning
FSU: Functional Swallowing Unit
HNC: Head and neck cancer
LR: Logistic regression
MBSS: Modified Barium Swallowing Studies
MDACC: MD Anderson Cancer Centre
NTCP: Normal tissue complication probability
OAR: Organ at risk
PCM: Pharyngeal constrictor muscle
PSS-HN: Performance Status Scale for Head and Neck cancer
RAD: Radiation-associated dysphagia
ResNet: Residual Network
UMCG: University Medical Centre Groningen
